# Public health diplomacy: summary of the methods and outcome of the 1st University of Memphis School of Public Health Diplomacy Summit

**DOI:** 10.3389/fpubh.2025.1564709

**Published:** 2025-04-11

**Authors:** Ashish Joshi, Laura Magana, Kun-Hsien Tsai, Diana Maddah, Kerry Mitchell, Diana Ruggiero, W. Bradley Hawkins, Shafik Dharamsi, Jihad Makhoul, Rodrigo Reis, So Yoon Kim, Wah Yun Low, Woldekidan Amde, Glory Nja, Michelle Jeu, Bridget Kelly, Bernard Saliba, Niharika Jha, Ramune Kalediene, Giada Scarpetti, Erica Kastrup, Rajendra Surenthirakumaran, Catherine Kane, William Yotive, Matthew Brown, Xinhua Yu, Gretchen Peterson, Beverly Tsacoyianis, Michael Arthur Ofori, Marian Levy, Stella Chirwa, Maryam Karimi, Kami Geron, Saloni Patel, Dorothy Biberman, Michelle Taylor, Aeryn Longuevan, Steve Shular, Tim K. Mackey

**Affiliations:** ^1^School of Public Health, University of Memphis, Memphis, TN, United States; ^2^Association of Schools and Programs of Public Health (ASPPH), Washington, DC, United States; ^3^Department of Public Health, National Taiwan University, Taipei, Taiwan; ^4^Public Health Department, Qatar University, Doha, Qatar; ^5^Department of Public Health and Preventive Medicine, St. George’s University, St. George, Grenada; ^6^Department of World Languages and Literatures, University of Memphis, Memphis, TN, United States; ^7^Institute for Medicine and Public Health, Vanderbilt University, Nashville, TN, United States; ^8^School of Public Health, University of North Texas Health Science Center, Fort Worth, TX, United States; ^9^Department of Health Promotion and Community Health, American University of Beirut, Beirut, Lebanon; ^10^People Health and Place Unit, Prevention Research Center, Washington University in St. Louis, St. Louis, MO, United States; ^11^Department of Medical Law and Ethics, Yonsei University, Seoul, Republic of Korea; ^12^Asia-Europe Institute, University Malaya, Kuala Lumpur, Malaysia; ^13^School of Public Health, University of the Western Cape, Bellville, South Africa; ^14^Department of Public Health, University of Calabar, Calabar, Nigeria; ^15^Early Start, School of Social Sciences, University of Wollongong, Wollongong, NSW, Australia; ^16^University of Technology Sydney, Sydney, NSW, Australia; ^17^Department of Health Management, Lithuanian University of Health Sciences, Kaunas, Lithuania; ^18^Department of Healthcare Management, Technische Universität Berlin, Berlin, Germany; ^19^CRIS Center for International Relations in Health of the Oswaldo Cruz Foundation, Rio de Janeiro, Brazil; ^20^Department of Community Medicine, University of Jaffna, Jaffna, Sri Lanka; ^21^Independent Researcher, Geneva, Switzerland; ^22^World Federation of United Nations Associations, Geneva, Switzerland; ^23^Global Health Policy and Data Institute, San Diego, CA, United States; ^24^Department of Sociology, University of Memphis, Memphis, TN, United States; ^25^Department of History, University of Memphis, Memphis, TN, United States; ^26^Shelby County Health Department, Shelbyville, IN, United States; ^27^Global Health Program, Department of Anthropology, UC San Diego, San Diego, CA, United States

**Keywords:** health diplomacy, public health diplomacy, global health governance, health policy, Global Health Security, public health competencies

## Abstract

Public health diplomacy addresses global challenges impacting societies, economies, the environment, and health by integrating foreign policy and development. The University of Memphis School of Public Health hosted a multistakeholder summit to identify strategies and competencies essential for effective public health diplomacy. A 3-day summit included 29 participants from 15 countries, representing the WHO, the World Federation of United Nations, and seven regional public health associations. An iterative human-centered design (HCD) approach and concept mapping were employed to facilitate discussions and generate actionable recommendations. Developed a working definition of Public Health Diplomacy emphasizing cross-disciplinary collaborations, communication, negotiation, and consensus building. Produced a 9-point action plan to establish a global framework, launch capacity-building initiatives, and institutionalize public health diplomacy as a public health discipline.

## Introduction

Global health issues, including the COVID-19 pandemic, climate change, misinformation, conflicts, and humanitarian crises, create complex health, economic, and geopolitical challenges (1). These crises disproportionately affect vulnerable populations, particularly women, girls, and children, underscoring the need for inclusive and equity-focused governance. Addressing these challenges effectively requires integrating public health into all policy areas through a “Health in All Policies” approach, which targets wider social determinants of health and promotes health equity across populations (2). Public health diplomacy is essential for fostering collaboration across governments, multilateral organizations, NGOs, academia, and the private sector, advancing global health priorities while contributing to shared development and security goals (3).

The COVID-19 pandemic exposed gaps in global health governance and highlighted the critical need for stronger and more equitable international cooperation (4). It demonstrated the duality of diplomacy during crises: one fostering solidarity and equity, and another seeking geopolitical advantage (5–7). Initiatives such as COVAX for vaccine access and the WHO Investment Round illustrate the potential of multilateral cooperation but also reveal inequities in implementation and access (8). Lessons from the pandemic stress the importance of combining formal diplomacy (e.g., health attachés and diplomats) with informal diplomacy, involving non-state actors like NGOs and private enterprises. Strengthening health diplomacy requires multidisciplinary approaches and cross-sector training to prepare professionals to navigate the sociopolitical and cultural complexities of global health (9–11).

This policy brief paper summarizes the outcomes of a first Public Health Diplomacy Summit organized by the University of Memphis, Public Health Diplomacy Lab, which aimed to define the field, emphasize its importance, and establish an action plan for education, capacity building, and the practice of public health diplomacy.

### Public Health Diplomacy Lab

The University of Memphis School of Public Health established the nation’s first Public Health Diplomacy Lab with an aim to bring together different disciplines and stakeholders—governments, NGOs, academics, and civic society to improve collaboration and coordination among all actors to promote health and well-being through the understanding of broad stakeholder engagement, consensus building, and negotiations.

### Public Health Diplomacy Summit

Growing numbers of countries have become increasingly more engaged in health policy, health governance, and health diplomacy. This includes recognition that even core health diplomacy actors, such as accredited diplomats, require more direct training, help, and active engagement with health experts. Hence, there is an urgent need to design and establish health diplomacy governance and partnership frameworks that can activate public health and other allies to collectively address global health challenges through traditional vehicles of policy mobilization and generating public support for health objectives. Recognizing this need for a new community of health diplomacy practitioners and advocates, the 2024 Public Health Diplomacy Summit, organized by the Public Health Diplomacy Lab, aimed to bring together stakeholders from diverse geographic, professional, and academic backgrounds to discuss the intersection of health and diplomacy and its role in advancing health equity.

**Figure 1:**
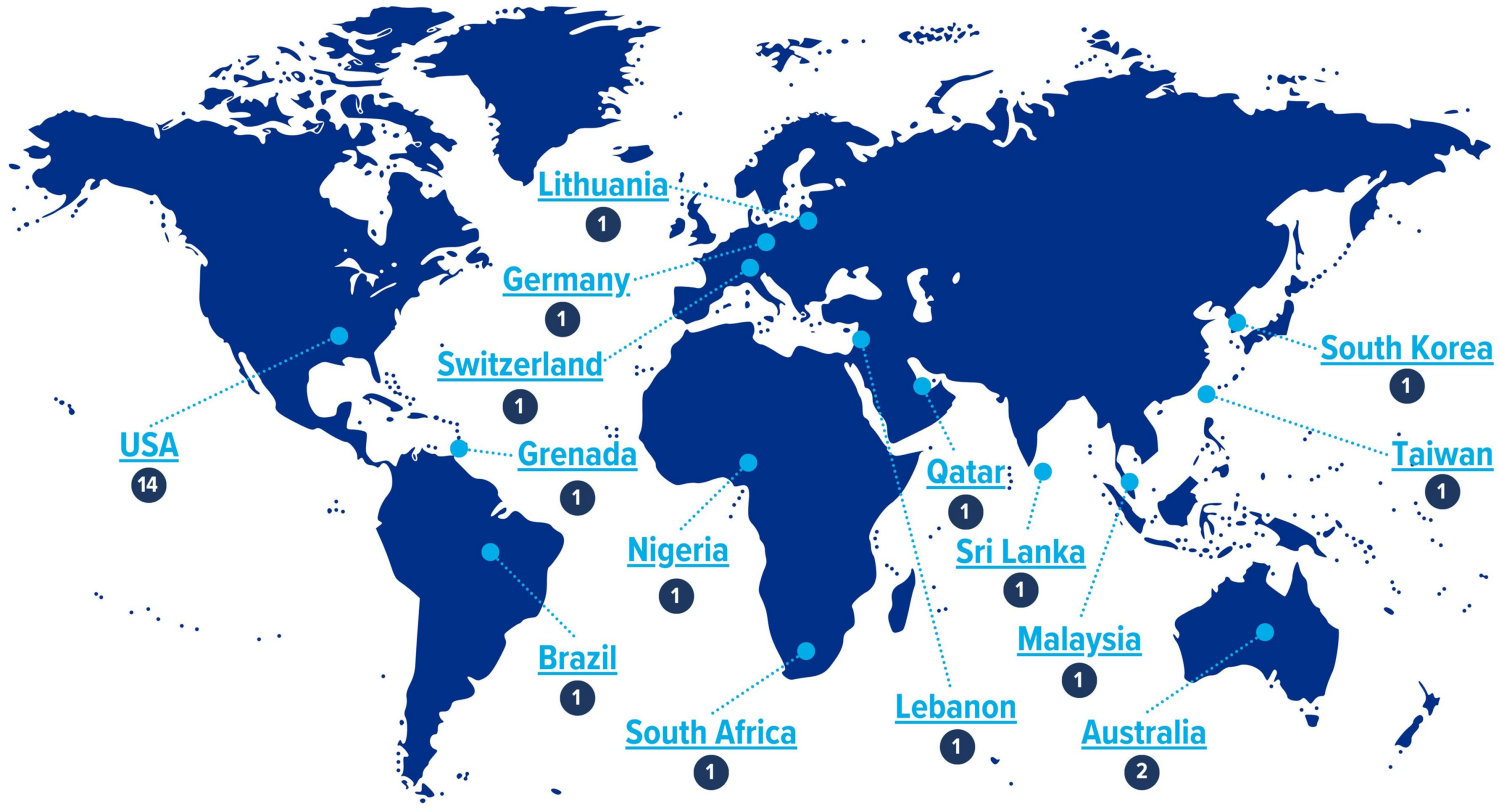
Participation of 15 countries (*n* = 29) representing 7 regional academic public health associations.

The lab, led by A Joshi, collaborated with the Association of Schools and Programs of Public Health (ASPPH) and reached out to all seven regional public health associations globally including Association of Schools and Programs in Public Health (ASPPH), Asia-Pacific Academic Consortium for Public Health (APACPH), Association of Schools of Public Health in Africa (ASPHA), Council of Academic Public Health Institutions Australia (CAPHIA), Association of Schools of Public Health in the European Region (ASPHER), Latin American Alliance for Global Health/Alianza Latin-American de Salud Global (ALSAG) and South-East Asia Public Health Education Institutions Network for representation in the summit (Figure 1). Diplomacy actors such as the WHO and the World Federation of United Nations Associations were also invited.

The summit featured 29 experts from 15 countries (Figure 1) with additional engagement from local public health agencies like the Shelby County Health Department. Of the 29 representatives, 28 attended in person, while one participated via Zoom due to unavoidable circumstances. The virtual attendee was fully integrated into the discussions through a hybrid meeting setup, ensuring real-time engagement and participation. This setup allowed for seamless interaction, with the participant contributing to discussions and accessing presentation materials alongside in-person attendees.

### Methodology of Public Health Diplomacy Summit

The summit employed an iterative human-centered design (HCD) approach (12) to engage attendees and gather insights on defining public health diplomacy and establishing it as a new field of study or core competency in public health. Held from September 25–27, 2024, the three-day event included both in-person and virtual sessions. On the first day, participants introduced themselves and shared their positions, perspectives, and objectives. The agenda and discussion questions were shared in advance. On the second day, participants were divided into five roundtable groups based on their academic, professional, and regional backgrounds. Facilitators led 40–45 min discussions on assigned topics, summarizing key insights for comparative analysis. To facilitate open and candid discussions, the Chatham House Rule was applied, meaning that while participants were free to use the information shared, the identity and affiliation of speakers could not be disclosed. This approach encouraged attendees to speak more freely, share honest perspectives, and engage in constructive debate without concern for political or professional repercussions. The third day focused on synthesizing these discussions, reaching consensus on core objectives, and developing actions to operationalize public health diplomacy, including building a global collaboration network.

Four key questions guided the discussions, (I) exploring the intersection of health and diplomacy, (II) required competencies for public health professionals, (III) resources for students engaging with health diplomacy, and (IV) global collaborations for education development. Using Dedoose software, the team applied thematic analysis to qualitative data, identifying four major discussion topics, 16 themes, and 47 sub-themes. These findings offer a detailed interpretation of public health diplomacy perspectives.

### Ethical considerations

Since the paper is based on observations of discussions at the summit and focuses on summarizing expert opinions under the Chatham House Rule, we did not seek approval from the Institutional Review Board. We did not collect any personal information from the participating experts.

## Results

Core concepts of the definition of health diplomacy and global health diplomacy were introduced to participants and used to develop a general gap analysis of what could formulate a working definition of “public health diplomacy.” The existing definitions were not inclusive enough of informal health diplomacy actors and not precise enough to the needs of public health professionals. Following are the themes generated from the participant discussions across the four discussion questions.

### 1. Health functions requiring diplomatic skills

Discussion one (Table 1) highlights how public health diplomacy can leverage multilateral collaboration to tackle global health challenges.

**Table 1:** Key themes and insights from discussion 1 of the public health diplomacy summit.

Theme	Subtheme	Key insights	Representative quotes
1. Health Diplomacy in Conflict Zone	Engagement in Crisis	Health diplomacy is critical in conflict zones where humanitarian crises limit access to healthcare. Diplomatic efforts engage governments and non-state actors to prioritize health.	*Health diplomacy is very important in reconstructing society… health is the most important issue in rebuilding the country.* *It’s their right… I do not care whether you are LGBT, migrant, or refugee, all of them have the right to health.* *We need to identify who we are interfacing with and what their needs are from the health community.* *Propose a special topic of health literacy diplomacy in existing conferences to attract new research and discussions.* *Young people trust influencers more than official health disseminators, making them critical for public health advocacy.* *Communicating ideas effectively with diverse audiences is probably the most important promise.* *We need to prioritize data protection and provide context for reporting case numbers.* *Cultural norms impact communication, not just language barriers but also regional expectations.*
	Neutrality of Health Diplomacy	Health diplomacy’s neutrality fosters collaboration by addressing shared health issues, transcending political or ideological divides.	
	Reconstruction and Sustainability	Post-conflict recovery relies on rebuilding sustainable health systems, addressing immediate needs and long-term infrastructure.	
2. Cultural Sensitivity and Inclusivity in Public Health	Cultural Barriers	Cultural norms can hinder public health measures when they conflict with traditional practices, making culturally tailored messaging essential. Additionally, adapting the delivery of services -such as involving community leaders or using culturally appropriate healthcare approaches - can improve acceptance and effectiveness.	
	Rights-Based Approach	A rights-based approach ensures inclusivity, equity, and respect for human rights in public health interventions.	
	Understanding Community Needs	Understanding communities requires assessing health challenges and socio-political contexts to ensure relevant interventions.	
3. Enhancing Visibility and Advocacy	Digital Advocacy and Communication	Digital tools enhance outreach, resource sharing, and dialogue, promoting health diplomacy across borders.	
	Engaging Diverse Stakeholders	Inclusion of diverse stakeholders, from grassroots organizations to policymakers, strengthens advocacy and decision-making processes.	
	Integration into Established Platforms	Integrating health diplomacy into existing forums increases visibility, attracting new research and practitioners.	
4. Engagement with Communities and Stakeholders	Feedback Mechanisms and Accountability	Transparent feedback ensures that communities’ inputs are valued and shared findings are accountable.	
	Leveraging social media and Influencers	Social media and influencers play a key role in public health advocacy, especially among younger demographics.	
	Stakeholder Involvement and Empowerment	Empowering marginalized stakeholders strengthens trust and ensures meaningful participation in health diplomacy initiatives.	
	Tailoring Communication for Audience Diversity	Effective communication strategies must align with target audiences’ cultural, linguistic, and social contexts.	
5. Data Security and Ownership in International Collaborations	Data Ownership Issues	Conflicts over data ownership in international collaborations can undermine trust and delay research.	
	Data Sharing Challenges	Reluctance to share data during crises, like COVID-19, hinders effective public health responses.	
	Ethical Considerations	Prioritize data privacy and informed consent to ensure fairness and transparency in health diplomacy.	
	Ethical Decision-Making in Diplomacy	Requires cultural and regional awareness to ensure interventions are appropriate and accepted.	

### 2. Required competencies and specific training to help public health professionals serve as public health diplomats

The second discussion (Table 2) prompt yielded three overall themes and several additional topics on the training, skills, and knowledge necessary for public health professionals to excel as public health diplomacy advocates.

**Table 2:** Key themes and insights from discussion 2 of the public health diplomacy summit.

Theme	Subtheme	Key insights	Representative quotes
1. Competencies for Health Diplomacy	Crisis Management and Negotiation Skills	Involves quick, evidence-based decision-making to address emergencies. Negotiation skills are crucial for reaching mutually beneficial agreements across diverse stakeholders.	*Knowing what to say, how to say it, and delivering evidence-based decisions during crises is essential*. *Public health diplomats need to understand data and translate it into terms different stakeholders can understand*. *If a public health diplomat speaks the native language, the impact would be even better.* *Leadership is about knowing when to engage, how, and with whom to act as a leader.* *You interact with individuals, not cultural stereotypes… cultural humility is key in diplomacy*. *It’s important to know how to talk to people from different levels, whether policymakers or marginalized groups*. *It’s not enough to have one training—this needs to be sustainable to build future generations.*
	Cross-Cultural Communication	It is vital for health diplomats to navigate diverse environments. It ensures health messages are understood, accepted, and acted upon by policymakers, healthcare providers, and communities.	
	Data Analysis and Knowledge Transfer	Ability to analyze and communicate data in accessible terms is a critical skill for public health diplomats, as it ensures that complex health information is understandable and actionable for diverse stakeholders.	
	Language and Plain Communication for Diverse Audiences	Multilingual skills and simplifying technical terms are crucial for public health diplomats to deliver culturally appropriate, accessible, and resonant health messages.	
	Leadership Training	Effective health diplomacy leadership involves navigating power dynamics, mastering risk communication, adapting to change, and engaging stakeholders at all levels.	
	Systems Thinking and Holistic Approaches	Systems thinking is a critical approach in health diplomacy, as it encourages professionals to view health systems as interconnected networks rather than isolated components.	
2. Soft Skills in Communication and Interpersonal Relationships	Building Trust and Empathy	Trust is a cornerstone of effective diplomacy, as it ensures that communication is heard, understood, and acted upon.	
	Cultural Competency	Understanding and respecting cultural differences is fundamental to avoiding misunderstandings and fostering inclusivity in health diplomacy.	
	Interpersonal Skills and Conflict Mediation	Diplomats in health diplomacy must be adept at navigating complex power dynamics, understanding how to engage with different stakeholders based on their roles, influence, and needs.	
3. Educational Integration of Public Health Diplomacy	Curriculum Development	Curriculum development in public health diplomacy must go beyond traditional health topics and integrate interdisciplinary approaches to address multifaceted nature of global health issues.	
	Experiential learning Opportunities	Practical training opportunities can play a crucial role in bridging the gap between academic learning and real-world application in public health diplomacy.	
	Sustainability of Diplomatic Training	Sustaining public health diplomacy training is essential to ensuring that future generations of diplomats are equipped with the skills needed to address evolving global health challenges.	

### 3. Existing resources available for public health students toward health diplomacy in a complex, rapidly changing, multilateral system, and the gaps for new resources

The discussion (Table 3) emphasizes building and enhancing resources, including formal training programs, global events, digital resource hubs, and regional training hubs. It also addresses the involvement of academic institutes, governments, and international organizations in strengthening the field.

**Table 3:** Key themes and insights from discussion 3 of the public health diplomacy summit.

Theme	Subtheme	Key insights	Representative quotes
1. Advancing Public Health Diplomacy Education and Training	Experiential Learning Opportunities	Apply classroom concepts to real-world situations, offering practical exposure to the complexities of public health diplomacy.	*Providing students with real-life field experience… enhances their understanding of diplomacy.* *Health is a human right, and this principle must guide all public health diplomacy initiatives.* *We need a uniform definition to build consensus and guide collaborative efforts* *Simulation training and internships help students develop negotiation and crisis management skills.* *We should invite NGOs and international organizations to be part of a working group for public health diplomacy.* *We need a resource hub that aggregates materials and ensures equitable access globally.* *A community of practice can sustain public health diplomacy through defined action plans and frameworks.*
	Mentorship and Peer Learning	Vital for bridging gap between academic learning and professional practice by connecting students with experienced diplomats who can offer guidance, insights, and real-world perspectives.	
	Rights-Based Approach to Health Equity	Ensure that all health initiatives are grounded in the fundamental principle that health is a human right, not a privilege.	
	Scope and Uniformity in Definition	Defining public health diplomacy clearly is essential for establishing a shared understanding of its scope, objectives, and strategies, which helps guide effective collaboration among various stakeholders	
2. Enhancing Public Health Diplomacy Education	Global Engagement Programs	Exchange programs expose students to diverse health systems, policies, and diplomatic practices across different countries.	
	Simulation and Training Programs	Prepare students for real-world public health diplomacy by teaching negotiation skills and providing controlled environments to practice tackling real-world challenges.	
3. Building Infrastructure for Collaboration and Learning	Global Networks and Partnerships	Collaboration between universities, NGOs, and governments plays a crucial role in advancing public health diplomacy by fostering shared learning, pooling resources, and enhancing collective impact.	
	Resource Hubs	Platform providing access to a wide range of materials, including training resources, case studies, research findings, and best practices in public health diplomacy.	
	Sustainability through Community of Practice	A community of practice (CoP) is a group of professionals who share a common interest or goal and work together to improve their practices through ongoing collaboration, learning, and knowledge exchange.	

### 4. Global and regional collaborations to develop resources for health diplomacy education

This discussion (Table 4) focused on the importance of partnerships, working groups, and initiatives to advance public health diplomacy. It included topics like mentorship and networking programs, collaboration with governments and NGOs, and creating opportunities for experiential learning.

**Table 4:** Key themes and insights from discussion 4 of the public health diplomacy summit.

Theme	Subtheme	Key insights	Representative quotes
1. Bridging Knowledge and Practice	Hands-On Experience	Field placements or real-world internships allow students to immerse themselves in the practical realities of global health issues.	*Public health diplomacy should integrate into existing academic fields to create continuity across disciplines.* *Partnerships between academic institutions and industries ensure diplomacy moves through and outside academia.* *Inviting vulnerable populations to decision-making tables fosters inclusivity and equity.* *We need to cultivate a participatory approach by involving communities at every stage of decision-making.*
	Integrative Curriculum Design	Equip students with interdisciplinary skills in public health diplomacy by combining public health, international relations, law, economics, and political science	
2. Collaboration and Partnership in Health Diplomacy	Academic-Community Partnerships	Vital for ensuring that public health diplomacy is not confined to academic institutions but also reaches and benefits local communities	
	Engagement with Vulnerable Populations	Ensure public health diplomacy addresses needs, fostering inclusive and equitable health interventions.	
	Government and NGO Roles	Key to global health diplomacy, with NGOs providing care, resources, and support in crises, while governments establish policies and diplomatic channels to enable these efforts.	
	Participatory Approach to Communities	Involves communities in decision-making, ensuring their needs shape policies and fostering ownership, empowerment, and trust for more effective, sustainable health initiatives.	

Participants also noted that currently, no established and widely accepted definition exists specifically for the public health diplomacy and so initial discussions were focused on establishing a working definition that was more aligned with the needs of informal health diplomacy actors and the actual practice of health diplomacy among public health communities and stakeholders. This task was assessed as a priority since the term “public health diplomacy” was associated with other established terms associated with the diplomacy such as “health diplomacy,” “global health diplomacy,” and/or “global diplomacy,” that have generally focused on the actions of core and multistakeholder diplomacy actors. Consensus was built around the definition of public health diplomacy, defined as:

*Public health diplomacy* is a multidisciplinary field that enables its practitioners to effectively communicate, facilitate, negotiate and build consensus using systems thinking, evidence based, community-informed approaches, based on equity-focused and human-centered values to improve health and well-being for all.

Following the discussions over the three days, the following 9-point action plan was established.

1. Participants proposed the establishment of a “*Global Public Health Diplomacy Working Group*” by inviting member schools and programs from ASPPH and GNAPH to review and build on the concept map to reflect and reconcile different perspectives and disciplines and the factors contributing to public health diplomacy.
2. Participants proposed the creation of “*Public Health Diplomacy capacity-building group*” globally that will work with key public health training and academic institutions across various regions to gather insights into the skills and competencies essential for public health students. The working group would also explore options and partnerships that could implement the review.
3. Participants agreed to Identify “*global and region-specific learning outcomes*” to prepare public health students to succeed in an interconnected and complex world through public health diplomacy. Participants assessed this task to be a priority.
4. Participants agreed to advance “*public health diplomacy by engaging decision-makers and stakeholders*” (including foreign services, ministries of health, academic institutes, civil society members, policy makers, and other international organizations like WHO and UN) in their geographic regions to create more awareness about the field.
5. Participants agreed to develop an online, interactive resource *hub* to disseminate global learning opportunities for Public Health Diplomacy Research, Innovation, Skills, and Experiential learning *(PHD-RISE)*.
6. Participants agreed to “*organize events*” to help raise awareness about public health diplomacy in the field of public health.
7. Participants agreed to “*analyze and share the region’s experiences, best practices and achievements*” in public health diplomacy across various communication outlets to further develop the field.
8. Participants agreed to “*expand regional and institutional partnerships*” in collaboration with the University of Memphis School of Public Health, ASPPH and GNAPH to advance public health diplomacy.
9. Participants agreed to *disseminate recommendations* from participants to their respective institutions to move forward.

These nine-point action plan is not intended to be sequential; rather, the actions should be pursued concurrently based on needs and capacities. Additionally, the numbering does not indicate prioritization, as implementation strategies may vary across different contexts.

## Conclusion

Public health diplomacy is a multidisciplinary field enabling practitioners to communicate, negotiate, and build consensus using equity-focused, evidence-based, and community-informed approaches to improve global health. Key recommendations included forming capacity-building groups, creating global learning outcomes, and establishing a resource hub for research, innovation, and skills development. Participants emphasized collaboration among governments, NGOs, academia, and communities to address health challenges through inclusive solutions.

Effective health diplomacy requires experiential learning, mentorship, and interdisciplinary education, but challenges like data sharing, cultural communication, and sustainability remain. Training in “practical diplomacy” equips global health students to navigate local and global challenges effectively.
