# Supplementary material for: Public health diplomacy: summary of the methods and outcome of the 1st University of Memphis School of Public Health Diplomacy Summit
Source: Front Public Health. 2025 Apr 11;13:1564709. doi: 10.3389/fpubh.2025.1564709 (PMC12023912; doi:10.3389/fpubh.2025.1564709)
Supplement: Supplementary file 1 [file Table_1.docx]

**Appendix 1. Summary of the themes and codes derived from the summit discussion session**

|  | Themes | **Sub Themes** |
| --- | --- | --- |
| Discussant One | | |
| What are the health functions that require diplomatic skills? | Theme One | |
|  | **Health Diplomacy in Conflict Zone** | **Engagement in Crisis** |
|  |  | **Neutrality of Health Diplomacy** |
|  |  | **Reconstruction and Sustainability** |
|  | Theme Two | |
|  | **Cultural Sensitivity and Inclusivity in Public Health** | Cultural barriers |
|  |  | Rights-based approach |
|  |  | **Understanding Community Needs** |
|  | Theme Three | |
|  | **Enhancing Visibility and Advocacy** | **Digital Advocacy and Communication** |
|  |  | **Engaging Diverse Stakeholders** |
|  |  | **Integration into Established Platforms** |
|  | Theme Four | |
|  | **Engagement with Communities and Stakeholders** | **Feedback Mechanisms and Accountability** |
|  |  | **Leveraging social media and Influencers** |
|  |  | **Stakeholder Involvement and Empowerment** |
|  |  | **Tailoring Communication for Audience Diversity** |
|  | Theme Five | |
|  | **Data Security and Ownership in International Collaborations** | **Data Ownership Issues** |
|  |  | **Data Sharing Challenges** |
|  |  | **Ethical Considerations** |
|  |  | **Ethical Decision-Making in Diplomacy** |
| Discussant Two | | |
| What competencies are required and what specific training will help public health professionals serve as Public Health Diplomats? | Theme One | |
|  | **Competencies for Health Diplomacy** | **Crisis Management &Negotiation Skills** |
|  |  | **Cross-Cultural Communication** |
|  |  | **Data Analysis and Knowledge Transfer** |
|  |  | **Language and Plain Communication for Diverse Audiences** |
|  |  | **Leadership Training** |
|  |  | **Systems Thinking and Holistic Approaches** |
|  | Theme Two | |
|  | **Soft Skills in Communication and Interpersonal Relationships** | **Building Trust and Empathy** |
|  |  | **Cultural Competency** |
|  |  | **Interpersonal Skills and Conflict Mediation** |
|  | Theme Three | |
|  | **Educational Integration of Public Health Diplomacy** | **Curriculum Development** |
|  |  | **Experiential learning Opportunities** |
|  |  | **Sustainability of Diplomatic Training** |
| Discussant Three | | |
| What are the existing resources available for public health students toward health diplomacy in a complex, rapidly changing, multilateral system, and what are the gaps for new resources? | Theme One | |
|  | **Advancing Public Health Diplomacy Education and Training** | **Experiential Learning Opportunities** |
|  |  | **Mentorship and Peer Learning** |
|  |  | **Rights-Based Approach to Health Equity** |
|  |  | **Scope and Uniformity in Definition** |
|  | Theme Two | |
|  | **Enhancing Public Health Diplomacy Education** | **Global Engagement Programs** |
|  |  | **Simulation and Training Programs** |
|  | **Theme Three** | |
|  | **Building Infrastructure for Collaboration and Learning** | **Global Networks and Partnerships** |
|  |  | **Resource Hubs** |
|  |  | **Sustainability through Community of Practice** |
| Discussant Four | | |
| Where do we start with global and regional collaborations to develop resources for health diplomacy education? | **Theme One** | |
|  | **Bridging Knowledge and Practice** | **Hands-On Experience** |
|  |  | **Integrative Curriculum Design** |
|  | **Theme Two** | |
|  | **Collaboration and Partnership in Health Diplomacy** | **Academic-Community Partnerships** |
|  |  | **Engagement with Vulnerable Populations** |
|  |  | **Government and NGO Roles** |
|  |  | **Participatory Approach to Communities** |
